# Joint and Independent Associations of Dietary Antioxidant Intakes With Advanced Stages in Older Patients With Cardiovascular‐Kidney‐Metabolic Syndrome

**DOI:** 10.1002/kjm2.70215

**Published:** 2026-05-13

**Authors:** Fu‐Shan Qiu, Jian‐Chao Qi, Mei‐Zhu Lin, Jun‐Hua Ke

**Affiliations:** ^1^ Department of Geriatric Rehabilitaton Rehabiliation Hospital Affiliated to Fujian University of Traditional Chinese Medicine Fuzhou Fujian China; ^2^ Fujian Key Laboratory of Rehabilitation Technology Fuzhou Fujian China; ^3^ Fuzhou University Affiliated Provincial Hospital Fuzhou Fujian China; ^4^ Department of Emergency Trauma Surgery Fujian Trauma Medicine Center, Fujian Key Laboratory of Emergency Medicine, Fujian Provincial Hospital Fuzhou China; ^5^ Department of Obstetrics and Gynecology Fujian Maternity and Child Health Hospital, College of Clinical Medicine for Obstetrics and Gynecology and Pediatrics, Fujian Medical University Fuzhou Fujian China

**Keywords:** cardiovascular‐kidney‐metabolic syndrome, composite dietary antioxidant index, NHANES, older adults, weighted quantile sum regression

## Abstract

This study explored the association between the composite dietary antioxidant index (CDAI) and cardiovascular‐kidney‐metabolic (CKM) progression in an older population. Using data from NHANES 2001–2020, we analyzed 4974 adults aged ≥ 60 years with CKM syndrome. The CDAI was calculated from the intake of six dietary antioxidants. Associations between the CDAI and its components and advanced CKM syndrome (Stages 3–4) were assessed using multivariable logistic regression, restricted cubic splines, piecewise logistic regression, and weighted quantile sum (WQS) regression. Participants in the highest CDAI quartile had lower odds of advanced CKM syndrome than those in the lowest quartile (OR = 0.728, 95% CI: 0.594–0.893). Restricted cubic spline analysis showed an L‐shaped association between CDAI and odds of advanced CKM syndrome, with an inflection point at 5.857; the inverse association was evident below this threshold and plateaued above it. Furthermore, the WQS regression model identified a protective combined effect of the six dietary antioxidants against advanced CKM syndrome, with vitamin A and vitamin C contributing the largest weights. Higher CDAI levels were associated with a lower risk of advanced CKM syndrome in older adults, with vitamins A and C emerging as the most influential components.

## 1 Introduction

Cardiovascular‐kidney‐metabolic (CKM) syndrome represents a complex interplay among metabolic dysregulation, chronic kidney disease (CKD), and cardiovascular disorders, with a particularly high prevalence in older populations [1, 2]. Epidemiological data show a marked age‐dependent increase in CKM prevalence, affecting up to 78.8% of adults aged ≥ 60 years at advanced Stages (3–4) [3]. This multisystem syndrome not only increases the risks of cardiovascular events, renal dysfunction, and metabolic complications, but also substantially raises all‐cause mortality and hospitalization rates [4, 5]. Therefore, early identification of CKM progression and timely intervention are critical for improving health outcomes in aging populations.

Accumulating evidence indicates that oxidative stress is a key mechanistic driver of CKM pathophysiology [6]. Dietary modulation is a promising therapeutic strategy, and nutritional antioxidants are potent regulators of oxidative stress and age‐related disease progression [7]. Previous studies have shown that specific antioxidants, such as vitamin E and flavonoids, have protective associations with cardiovascular and renal complications, whereas antioxidant deficiency may accelerate metabolic deterioration [8, 9]. The composite dietary antioxidant index (CDAI), which integrates six key micronutrients, vitamins A, C, and E, selenium, zinc, and carotenoids, provides a comprehensive marker of systemic antioxidant capacity [10]. Although observational studies have associated higher CDAI values with lower CKD incidence and mortality, particularly in older adults and patients with diabetes, important knowledge gaps remain [11, 12]. Current epidemiological evidence on dietary intervention in CKM is limited; CKM stage transitions have not been adequately assessed, and nutrient–nutrient and nutrient‐disease interactions have not been sufficiently considered.

To address these limitations, we used NHANES 2001–2020 data to examine the association between CDAI and CKM progression from Stages 1–2 to Stages 3–4 in adults aged ≥ 60 years. Using advanced statistical approaches, we aimed to: (1) quantify the independent predictive value of CDAI for advanced CKM syndrome, and (2) identify the antioxidant components with the greatest contribution. As the first nationally representative study to evaluate comprehensive antioxidant patterns in CKM progression, our findings provide evidence‐based insights for dietary interventions in this high‐risk population and help bridge critical gaps between nutritional science and CKM stage management.

## 2 Material and Methods

### 2.1 Study Population

The National Health and Nutrition Examination Survey (NHANES) is an ongoing cross‐sectional surveillance program conducted by the National Center for Health Statistics (NCHS), Centers for Disease Control and Prevention [13]. It uses a stratified, multistage probability cluster sampling design to recruit a nationally representative sample of approximately 5000 noninstitutionalized US residents each year. Data collection includes demographic characteristics, physical examination findings, laboratory results, and dietary data obtained through 24‐h recalls. All participants provided informed consent for survey participation and subsequent analysis of deidentified data. Survey protocols, data collection procedures, and public datasets are available at https://wwwn.cdc.gov/nchs/nhanes/.

This observational study adhered to the Strengthening the Reporting of Observational Studies in Epidemiology (STROBE) guidelines. We analyzed six consecutive NHANES cycles (2001–2020), including participants aged ≥ 60 years with CKM syndrome and complete data. Exclusion criteria were: (1) incomplete 24‐h dietary recall data, (2) missing CKM syndrome diagnostic components, or (3) unavailable covariate data (including body mass index, poverty‐income ratio, and other key variables). Following exclusions and quality control, the final analytical cohort comprised 4974 eligible participants (Figure S1).

### 2.2 CDAI Measurement

NHANES collects dietary intake data using two nonconsecutive 24‐h dietary recalls. The first interview is conducted in person at the Mobile Examination Center (MEC), followed by a telephone interview 3–10 days later. Dietary supplement use is assessed separately through a dietary supplement questionnaire. To comprehensively evaluate antioxidant exposure, we calculated the average total daily nutrient intake for each participant by combining nutrients derived from food and beverages with those derived from dietary supplements. The CDAI was then calculated using a modified method described by Wright et al. [14]. This index sums the standardized total daily intakes (z scores) of six dietary antioxidants: zinc, selenium, carotenoids, vitamin A, vitamin C, and vitamin E. Standardization was performed by subtracting the population mean and dividing by the standard deviation (SD) for the total intake of each nutrient.

### 2.3 Definition of CKM Syndrome

Information was collected through standardized questionnaires and physical examinations. Blood and random urine samples were analyzed in a central laboratory. We calculated glomerular filtration rate using the 2021 nonracial Chronic Kidney Disease Epidemiology Collaboration creatinine equation (Table S1) [15].

CKM syndrome was defined as the coexistence of subclinical or clinical cardiovascular disease (CVD), CKD, and metabolic disease (detailed definitions are provided in Table S2). Clinical CVD was defined as a history of chronic heart failure, coronary heart disease, heart attack, or stroke. Subclinical CVD was defined as a 10‐year CVD risk of ≥ 20% or high‐risk CKD. We calculated the predicted 10‐year CVD risk using a simplified CKM risk algorithm that incorporates age, sex, smoking, blood pressure, cholesterol, diabetes, kidney function, antihypertensive medication use, and statin use (Table S2). According to the Kidney Disease: Improving Global Outcomes classification, we stratified CKD risk using different thresholds for glomerular filtration rate (< 30, 30–44, 45–59, and ≥ 60 mL/min/1.73 m^2^) and urine albumin‐to‐creatinine ratio (< 30, 30–299, and ≥ 300 mg/g). In this study, CKD was defined as moderate‐ or high‐risk CKD. Metabolic disorders included overweight or obesity, abdominal obesity, prediabetes, diabetes, hypertension, dyslipidemia, and metabolic syndrome.

Considering the varying clinical severity of different forms of CKM syndrome, we chose to classify participants into four CKM stages: Stage 1, involving only obesity or prediabetes; Stage 2, involving at least one other metabolic disorder or CKD; Stage 3: subclinical CVD with metabolic disorders or CKD; Stage 4, clinical CVD with metabolic disorders or CKD [16]. Detailed staging criteria are outlined in Table S4. In this study, CKM syndrome staging ≥ Stage 1 was considered CKM syndrome, and advanced CKM syndrome staging was defined as CKM syndrome Stages 3 and 4, as these stages indicate that participants have or are at high risk for cardiovascular disease (Table S3).

### 2.4 Covariates

Based on previous studies [11, 17, 18] and a directed acyclic graph (DAG; Figure S2), we adjusted for the following confounders: age; sex (male/female); race/ethnicity; education; poverty‐income ratio (PIR); body mass index (BMI); smoking status based on serum cotinine concentration (nonsmoker, < 0.011 ng/mL; passive smoking, 0.011–9.999 ng/mL; active smoking, ≥ 10 ng/mL); alcohol intake (nondrinker, 0 g/day; low to moderate, men: 0.1–27.9 g/day and women: 0.1–13.9 g/day; heavy, men: ≥ 28 g/day and women: ≥ 14 g/day); physical activity (self‐reported vigorous or moderate recreational activity: yes/no); and self‐reported comorbidities, including hypertension (yes/no) and diabetes (yes/no).

### 2.5 Statistical Analysis

Continuous variables were assessed for normality, and all were nonnormally distributed. Accordingly, continuous variables were summarized as medians and interquartile ranges (IQRs), whereas categorical variables were presented as frequencies and weighted percentages. Differences across CDAI quartiles were evaluated using the Kruskal–Wallis test for continuous variables and the chi‐squared test for categorical variables. To examine the association between the CDAI score and its individual components and odds of advanced CKM syndrome, we performed multivariable logistic regression analyses. Variables with statistical significance in univariable analyses (two‐tailed *p* < 0.05) were included in the multivariable models. Both categorical and continuous forms of the CDAI score were analyzed. In the categorical analysis, CDAI was divided into quartiles, with the lowest quartile as the reference group. For trend analyses, ordinal values were assigned to CDAI quartiles and entered into regression models.

Three models were developed: the crude model (unadjusted); Model 1, adjusted for age, sex, and race; and Model 2, further adjusted for BMI, PIR, education level, smoking status, alcohol consumption, hypertension, diabetes, and physical activity. All analyses accounted for NHANES survey weights. To further examine the association between CDAI and its components and advanced CKM syndrome, generalized additive models (GAMs) with smoothed curve fitting using penalized splines were applied. When nonlinear relationships were detected, a recursive algorithm was used to identify inflection points. Piecewise logistic regression models were then fitted on either side of the inflection point [19].

Additionally, we performed weighted quantile sum (WQS) regression using the gWQS R package. WQS regression is a method for assessing the joint and individual effects of multiple correlated exposures. The dataset was randomly divided into a training set (40%) and a validation set (60%), and the training set was subjected to 400 bootstrap iterations [20]. The overall effect of the mixture of six dietary antioxidants and the weight of each component, which indicates the relative contribution of each antioxidant to the risk of advanced CKM syndrome, was estimated. The weights were constrained to sum to one, and any antioxidant with a weight > 1/6 was considered a major contributor to the observed association. Covariates included in the WQS model were the same as those used in the fully adjusted multivariable logistic regression model.

To assess the robustness of our findings, three sensitivity analyses were conducted. First, we re‐examined the association between CDAI and the odds of advanced CKM syndrome using multivariable logistic regression after excluding extreme outliers, defined as CDAI values beyond three standard deviations from the mean (i.e., < mean−3SD or > mean + 3SD). Second, subgroup analyses were performed to assess potential effect modification by key covariates. Interaction terms between CDAI and each stratification variable were added to separate regression models, and Wald tests were used to calculate *p* values for interaction. Third, to evaluate the relative importance of different predictors of advanced CKM syndrome, we used the eXtreme Gradient Boosting (XGBoost) machine learning algorithm [21]. All statistical analyses were conducted using R software (version 4.2.1). Two‐sided *p* < 0.05 was considered statistically significant.

## 3 Results

### 3.1 Participant Characteristics

Participants included in the analysis were categorized into four groups based on CDAI quartiles, and their weighted baseline characteristics are presented in Table 1. A total of 4974 individuals aged 60 years and older were included, of whom 43.7% had advanced CKM syndrome. The mean age of the cohort was 70.02 ± 7.12 years, and 49.9% were male and 50.1% were female. The CDAI quartiles were defined as follows: Q1 (−7.80 to −2.33), Q2 (−2.33 to −0.42), Q3 (−0.42 to 1.96), and Q4 (1.96 to 31.51). Among all participants, 12.1% were current smokers, 79.7% had hypertension, and 35.2% had diabetes. Compared with the other groups, participants in the highest CDAI quartile were more likely to be younger, non‐Hispanic White, have a lower BMI, have higher socioeconomic status, and have greater educational attainment. CDAI scores were also higher among individuals who reported alcohol consumption and those who had never smoked. As CDAI increased, the proportions of physically active individuals and those without hypertension or diabetes also increased (*p* < 0.05).

**TABLE 1:** Baseline characteristics of the study population by quartiles of composite dietary antioxidant index (CDAI score).

Characteristics	Overall (*N* = 4974)	Quartiles of CDAI score				*p*
		Q1 (*N* = 1244)	Q2 (*N* = 1243)	Q3 (*N* = 1242)	Q4 (*N* = 1245)	
Age, years, median (IQR)	69 (64, 76)	70 (64, 77)	70 (64, 76)	69 (63, 76)	68 (63, 75)	0.003
Gender (%)						0.008
Male	2490 (50.1)	598 (48.1)	643 (51.7)	660 (53.1)	589 (47.3)	
Female	2484 (49.9)	646 (51.9)	600 (48.3)	582 (46.9)	656 (52.7)	
Race (%)						< 0.001
Non‐Hispanic white	2780 (55.9)	603 (48.5)	666 (53.6)	741 (59.7)	770 (61.8)	
Non‐Hispanic black	881 (17.7)	286 (23.0)	220 (17.7)	189 (15.2)	186 (14.9)	
Mexican American	675 (13.6)	189 (15.2)	197 (15.8)	155 (12.5)	134 (10.8)	
Others	638 (12.8)	166 (13.3)	160 (12.9)	157 (12.6)	155 (12.5)	
Education (%)						< 0.001
Lower than high school	1430 (28.7)	527 (42.4)	375 (30.2)	299 (24.1)	229 (18.4)	
High school or equivalent	1242 (25.0)	341 (27.4)	320 (25.7)	310 (25)	271 (21.8)	
College or above	2302 (46.3)	376 (30.2)	548 (44.1)	633 (51)	745 (59.8)	
PIR (%)						< 0.001
0.00–1.30	1254 (25.2)	436 (35.0)	340 (27.4)	262 (21.1)	216 (17.3)	
1.31–3.50	2180 (43.8)	580 (46.6)	549 (44.2)	549 (44.2)	502 (40.3)	
> 3.51	1540 (31.0)	228 (18.4)	354 (28.4)	431 (34.7)	527 (42.4)	
BMI (%)						0.014
Normal < 25	1218 (24.5)	313 (25.2)	289 (23.2)	271 (21.8)	345 (27.7)	
Overweight 25–30	1896 (38.1)	483 (38.8)	472 (38.0)	474 (38.2)	467 (37.5)	
Obese > 30	1860 (37.4)	448 (36.0)	482 (38.8)	497 (40.0)	433 (34.8)	
Physical activity (%)						< 0.001
No	2751 (55.3)	819 (65.8)	719 (57.8)	650 (52.3)	563 (45.2)	
Yes	2223 (44.7)	425 (34.2)	524 (42.2)	592 (47.7)	682 (54.8)	
Smoking status (%)						< 0.001
Nonsmoking	2332 (46.9)	533 (42.8)	615 (49.5)	567 (45.7)	617 (49.6)	
Passive smoking	2037 (41.0)	484 (38.9)	494 (39.7)	538 (43.3)	521 (41.8)	
Active smoking	605 (12.1)	227 (18.3)	134 (10.8)	137 (11.0)	107 (8.6)	
Alcohol drinking (%)						< 0.001
Nondrinking	767 (15.4)	243 (19.5)	189 (15.2)	167 (13.4)	168 (13.5)	
Low to moderate drinking	1453 (29.2)	427 (34.3)	378 (30.4)	351 (28.3)	297 (23.9)	
Heavy drinking	2754 (55.4)	574 (46.2)	676 (54.4)	724 (58.3)	780 (62.6)	
Hypertension (%)						0.030
No	1008 (20.3)	225 (18.1)	241 (19.4)	260 (20.9)	282 (22.7)	
Yes	3966 (79.7)	1019 (81.9)	1002 (80.6)	982 (79.1)	963 (77.3)	
Diabetes (%)						< 0.001
No	3221 (64.8)	760 (61.1)	776 (62.4)	834 (67.1)	851 (68.4)	
Yes	1753 (35.2)	484 (38.9)	467 (37.6)	408 (32.9)	394 (31.6)	

*Note:* The continuous variables were analyzed by unweighted Wilcoxon test, expressed by the median (IQR); the unweighted chi‐squared test was used to analyze the categorical variables, expressed by the *n* (%).

Abbreviations: BMI, body mass index; PIR, poverty‐to‐income ratio.

### 3.2 Distribution and Concentration of CDAI and Its Components

Table S4 presents the distribution and concentrations of the CDAI and its individual components among US adults from NHANES 2001–2020. The mean CDAI score was 0.18, with values ranging from −7.80 to 31.51 in the study population. Percentile values indicated substantial variability: the 5th percentile was −4.59, the median (50th percentile) was −0.42, and the 95th percentile was 6.77. Spearman correlation coefficients presented in Figure S3 illustrate the relationships among the CDAI components. These correlations ranged from weak, for example, *r* = 0.26 between selenium and total carotenoids, to strong, for example, *r* = 0.63 between zinc and selenium, indicating different degrees of co‐consumption or shared metabolic patterns among antioxidants.

### 3.3 Association Between the CDAI and ItsSubcomponent Scores and Advanced CKM Syndrome

Table 2 presents the multivariable logistic regression results for the association between CDAI quartiles and the odds of advanced CKM syndrome among older adults. A significant inverse association was observed across all models, with higher CDAI levels associated with lower odds of advanced CKM syndrome. Notably, participants in the highest quartile (Q4) showed the strongest inverse association compared with those in the lowest quartile (Q1). In the fully adjusted model, Q4 was associated with 27.2% lower odds of advanced CKM syndrome (OR = 0.728, 95% CI: 0.594–0.893, *p* < 0.001). Consistent with these findings, Table S5 presents regression analyses for individual dietary antioxidant micronutrients. Four nutrients, vitamins A and C, selenium, and carotenoids, showed consistent and significant inverse associations with the odds of CKM syndrome progression across all models.

**TABLE 2:** Weighted logistic regression analysis of quartiles of composite dietary antioxidant index (CDAI) with odds of advanced CKM syndrome among older adults in NHANES 2001–2020.

	Crude		Model 1		Model 2	
	OR (95% CI)	*p*	OR (95% CI)	*p*	OR (95% CI)	*p*
Per 1 unit increase	0.952 (0.937, 0.967)	< 0.001	0.957 (0.940, 0.975)	< 0.001	0.984 (0.965, 1.003)	0.105
Quartiles of CDAI						
Quartile 1	Reference				Reference	
Quartile 2	0.776 (0.663, 0.909)	0.002	0.764 (0.639, 0.913)	0.003	0.833 (0.685, 1.013)	0.067
Quartile 3	0.686 (0.586, 0.804)	< 0.001	0.676 (0.564, 0.809)	< 0.001	0.767 (0.628, 0.936)	0.009
Quartile 4	0.547 (0.466, 0.642)	< 0.001	0.558 (0.465, 0.670)	< 0.001	0.728 (0.594, 0.893)	0.002
*p* for trend	< 0.001		< 0.001		0.002	

*Note:* Model 1 was adjusted for age, gender, race/ethnicity; Model 2 was adjusted as model 1 plus education levels, PIR, BMI, smoking status, physical activity, alcohol intake, hypertension, and diabetes.

Abbreviations: CI, confidence interval; CKM, cardiovascular‐kidney‐metabolic; NHANES, National Health and Nutrition Examination Survey; OR, odds ratio.

To visualize the association between CDAI and advanced CKM syndrome, a smoothed curve was fitted using GAMs based on the covariates in Model 2. As shown in Figure 1, a nonlinear L‐shaped relationship was observed. Threshold effect analysis further identified a turning point at a CDAI value of 5.857 (log‐likelihood ratio test, *p* = 0.001). Below this threshold, each one‐unit increase in CDAI was associated with a 4.9% reduction in the odds of advanced CKM syndrome. Above the threshold, however, the association plateaued, suggesting that further increases in CDAI did not provide additional protective benefit. These findings are detailed in Table S6. Figure S4 provides additional evidence through multivariable‐adjusted restricted cubic spline analysis of individual dietary antioxidant micronutrients in relation to advanced CKM syndrome based on NHANES 2001–2020 data.

**FIGURE 1:**
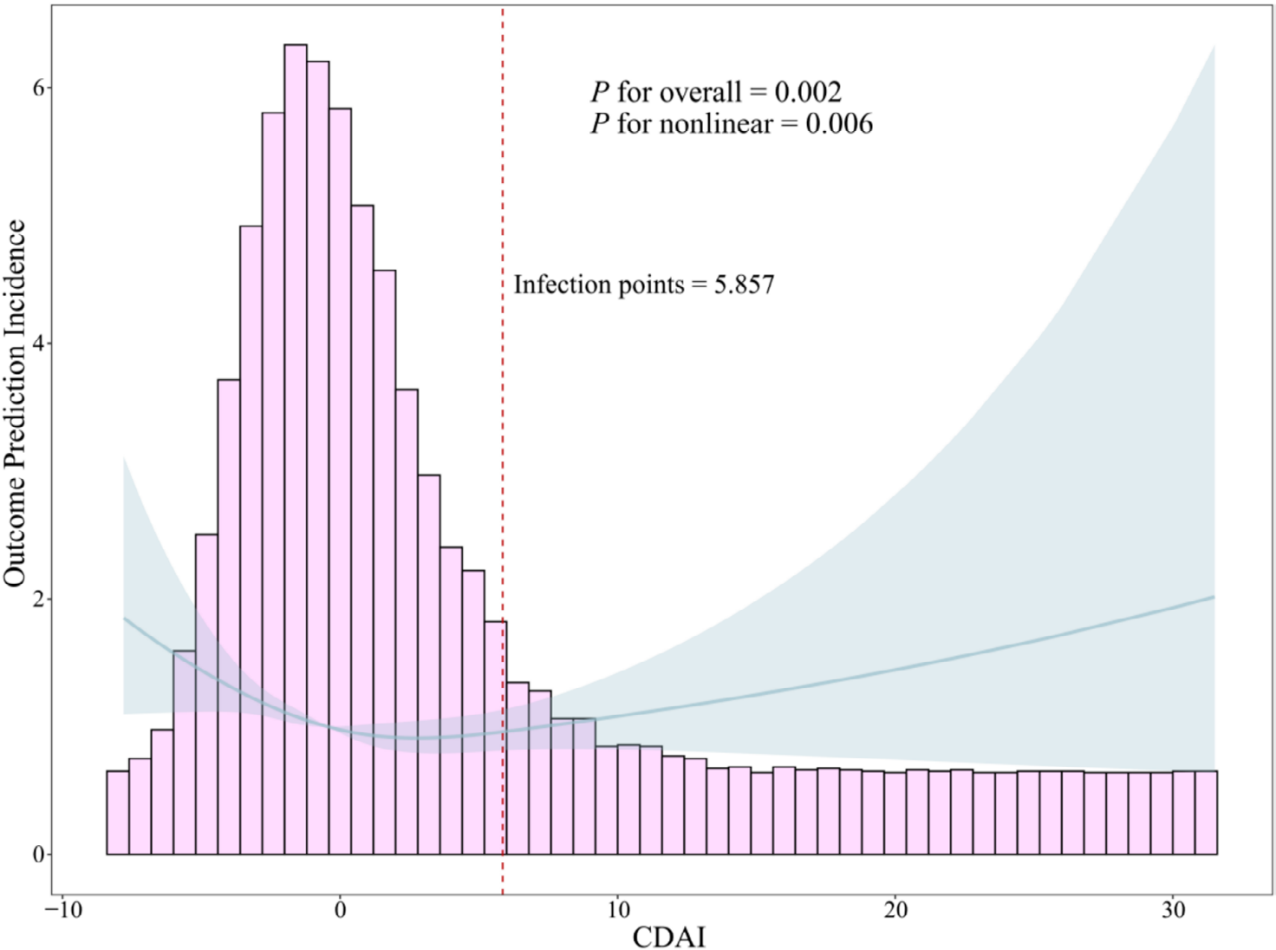
Restricted cubic spline (RCS) analysis with multivariate‐adjusted associations between CDAI and odds of advanced CKM syndrome in older adults. CDAI, composite dietary antioxidant index; CKM, cardiovascular‐kidney‐metabolic. Model was adjusted for age, gender, race/ethnicity, education levels, PIR, BMI, smoking status, physical activity, alcohol intake, hypertension, and diabetes.

### 3.4 Multiantioxidants Mixing Effect and the Odds of Advanced CKM Syndrome

To further investigate the combined effects of antioxidant micronutrients on the risk of advanced CKM syndrome, we constructed a WQS regression model. Joint exposure to antioxidant components was significantly associated with a lower risk of advanced CKM syndrome (OR = 0.854, 95% CI: 0.757–0.963). The estimated weights for the six antioxidants included in the model are presented in Figure 2. Among these, vitamin A contributed the most to the overall effect (weight = 0.339), followed by vitamin C (weight = 0.261) and selenium (weight = 0.218), indicating that these nutrients contributed most strongly to the observed protective association.

**FIGURE 2:**
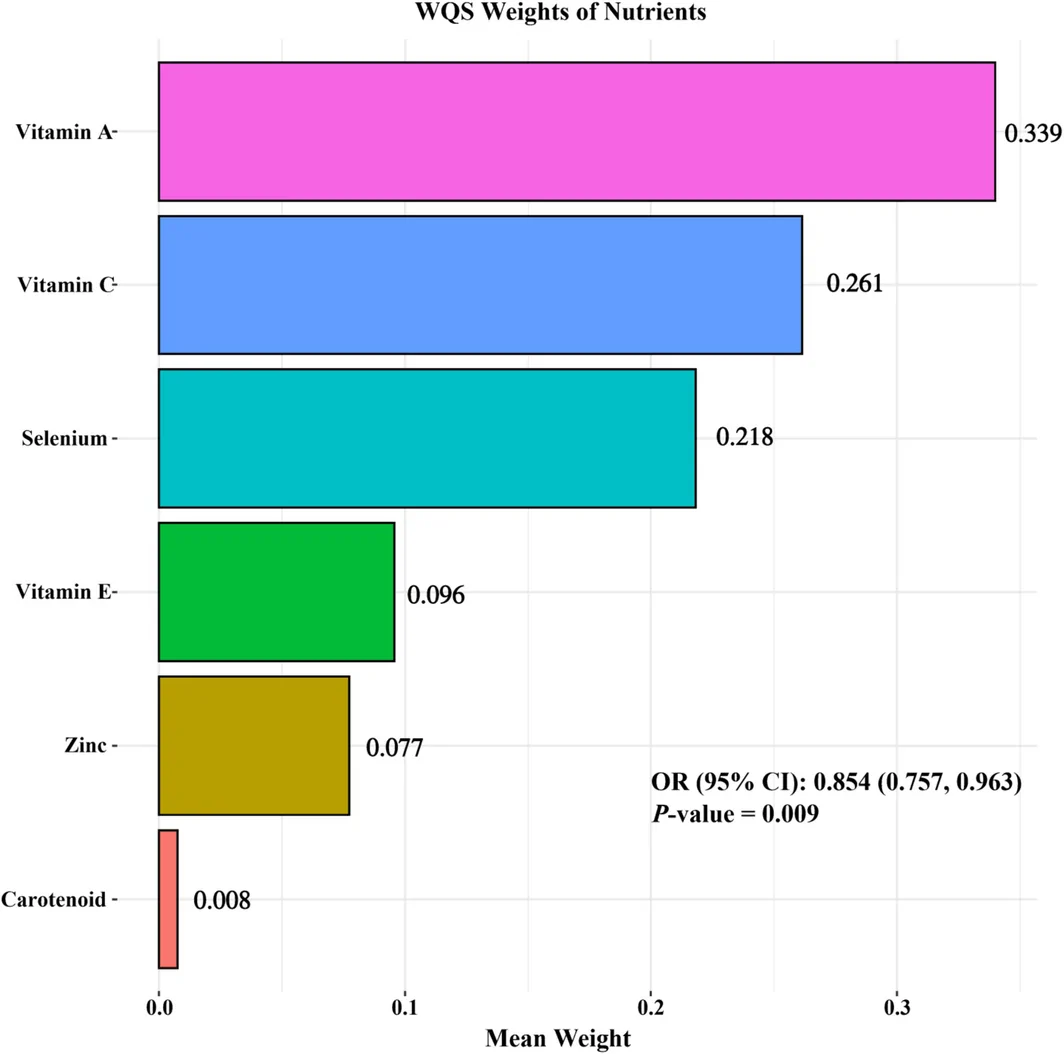
Weights from weighted quantile sum regression (WQS) for the combined intake of six dietary antioxidants in relation to odds of advanced CKM syndrome in older adults. Model was adjusted for age, gender, race/ethnicity, education levels, PIR, BMI, smoking status, physical activity, alcohol intake, hypertension, and diabetes.

### 3.5 Sensitivity Analyses

Sensitivity analyses were conducted using weighted multivariable logistic regression after excluding extreme CDAI values. Consistent with the main findings, all results showed an inverse association between CDAI and advanced CKM syndrome in both the fully adjusted and unadjusted models, as shown in Table S7. In addition, subgroup analysis (Table S8) showed that the association between CDAI and advanced CKM syndrome was robust across different population groups. Stratification factors did not materially modify this association (*p* for interaction > 0.05). Finally, to assess the importance of CDAI relative to other clinical factors, we used an XGBoost machine learning model to determine the contribution of selected variables to the prediction of advanced CKM syndrome. The four most influential factors were age, sex, diabetes, and CDAI, based on their contributions to the XGBoost model (Figure 3A). SHAP (SHapley Additive exPlanations) analysis further quantified the consistent contribution of CDAI to the model predictions (mean absolute SHAP value = 0.476; Figure 3B). These machine learning results support the robustness of the association between CDAI and the odds of advanced CKM syndrome. Overall, these findings suggest that CDAI is a significant and stable predictor in the multifactorial context of advanced CKM syndrome.

**FIGURE 3:**
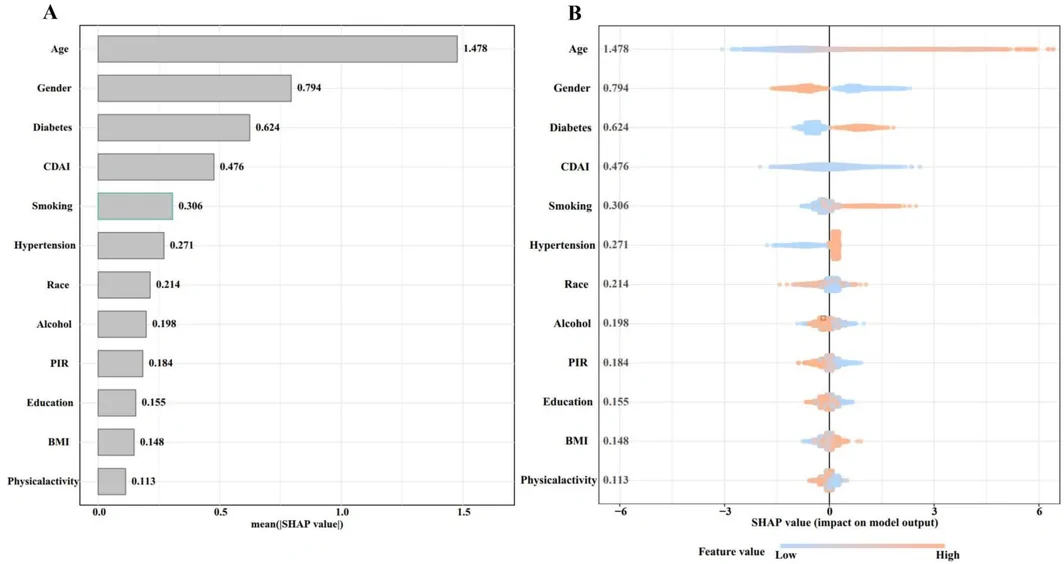
Clarification of machine learning models through SHAP summary and dependence plots (A, B) Importance matrix and SHAP summary plot display the contributions of all variables to the XGBoost model. Each line represents a feature variable, with the *x*‐axis corresponding to the SHAP value. Red dots indicate higher feature values, while blue dots indicate lower feature values. SHAP values higher than zero indicate higher odds of advanced CKM syndrome in older adults. Model was adjusted for age, gender, race/ethnicity, education levels, PIR, BMI, smoking status, physical activity, alcohol intake, hypertension, and diabetes.

## 4 Discussion

Using nationally representative data from NHANES 2001–2020, we investigated the association between the CDAI and the progression of CKM syndrome in a cohort of 4974 older adults. Fully adjusted models showed a significant inverse association between CDAI and advanced CKM syndrome, suggesting a protective role of dietary antioxidants in disease progression. Notably, restricted cubic spline analysis indicated a nonlinear L‐shaped relationship between CDAI and advanced CKM syndrome, with an inflection point at 5.857. WQS regression further supported these findings, showing that higher overall antioxidant intake was associated with lower odds of advanced CKM syndrome, with vitamins A and C contributing most prominently. To our knowledge, this is the first US population‐based study to comprehensively assess the association between CDAI and advanced CKM syndrome across disease stages.

A central pathophysiological mechanism of CKM syndrome is multiorgan interactive injury driven by oxidative stress. Excess adipose tissue can induce insulin resistance, promote the release of inflammatory cytokines such as TNF‐α and IL‐1β, and lead to vascular dysfunction, thereby establishing a vicious cycle of metabolic disorder, renal injury, and cardiovascular events [21, 22]. The CDAI integrates six major dietary antioxidants, vitamins A, C, and E, zinc, selenium, and carotenoids, and provides a systematic assessment of overall dietary antioxidant capacity [23, 24]. Previous studies have demonstrated a broad protective role of CDAI across different stages of CKM progression. During the metabolic risk stage, elevated CDAI levels are significantly associated with lower risks of general and abdominal obesity, with stronger effects in women and individuals in the metabolic compensation stage [25]. However, several gaps remain in the current literature. First, evidence is lacking regarding the protective effect of CDAI across the full cascade from abdominal obesity to organ damage to heart or kidney failure. Second, although a threshold of −2.99 has been identified for stroke risk [26], the optimal dietary intervention threshold for the entire CKM trajectory remains unclear. Therefore, this study aimed to comprehensively evaluate the protective association of CDAI with CKM syndrome and to use restricted cubic spline models to identify a potential intervention threshold, thereby providing a scientific basis for stratified nutritional interventions in high‐risk populations.

In our WQS analysis, preformed vitamin A had a higher weight (0.339) than β‐carotene, likely reflecting inherent physiological constraints in carotenoid metabolism. The bioconversion of β‐carotene to retinol is highly variable and often inefficient, with weight‐based conversion ratios ranging from 3.8:1 to 28:1 [27, 28]. In addition, unlike directly bioavailable preformed vitamin A, β‐carotene absorption is limited by food matrices and feedback regulation, and it may exert biological effects independent of its provitamin A activity [29]. Thus, these nutrients represent different dietary exposures with different metabolic effects in this population. Selenium also showed a notable weight (0.218) in the WQS model, second only to vitamins A and C, suggesting potential relevance in CKM syndrome. As an essential trace element with antioxidant and immunomodulatory properties, selenium has been implicated in renal protection [30, 31]. However, in our study, the protective association of selenium did not show a clear dose‐dependent pattern. Specifically, selenium intake in the second (Q2) and third (Q3) quartiles was not significantly associated with CKM risk, and a significant inverse association was observed only in the highest quartile (Q4). Furthermore, restricted cubic spline analysis showed an L‐shaped relationship between selenium intake and CKM risk, although both the overall and nonlinear *p* values were > 0.05, indicating insufficient statistical evidence for this association. This nonlinear pattern may reflect the well‐documented U‐shaped dose–response relationship of selenium, in which both insufficient and excessive intake may increase disease risk. These findings suggest that although selenium may have protective potential, recommending higher intake without a clearly defined safe range may not provide the expected benefit and may even pose risks [32]. Future studies should examine the optimal selenium intake range and its interactions with other antioxidant nutrients.

It should also be clarified that the WQS weights for each antioxidant reflect their relative contribution to the joint protective association of the overall mixture with advanced CKM syndrome. These weights should not be interpreted as direct measures of the individual or causal protective effect of each nutrient in isolation [20]. For instance, although vitamin A and vitamin C showed the highest weights in the mixture model, this finding indicates that within the complex dietary matrix, they were the primary contributors to the observed combined association. It does not necessarily mean that increasing vitamin A or vitamin C intake alone would produce the greatest risk reduction compared with the other nutrients. This interpretation is consistent with mixture analysis methodology, which is intended to reflect real‐world dietary patterns in which nutrients are consumed together and may interact.

The precise mechanistic association between CDAI and advanced CKM syndrome remains unclear, although some evidence suggests that higher antioxidant levels contribute to lower odds of CKM syndrome. In metabolic tissues such as adipose tissue and liver, oxidative stress disrupts mitochondrial respiration, impairs insulin signaling, worsens dyslipidemia, and accelerates renal injury through glomerular hyperfiltration and fibrosis. This is consistent with the elevated ACR and reduced eGFR observed in the low‐CDAI group in previous studies [33]. As an external factor, diet modulates plasma redox status and protects the body against reactive oxygen and nitrogen species. To maintain redox balance, antioxidants scavenge oxidants and thereby prevent oxidative stress [34]. Exogenous antioxidant intake has been shown to reduce inflammation, atherosclerosis, insulin resistance, and oxidative stress in patients with CVD, CKD, and metabolic syndrome [35]. Our findings identified an L‐shaped relationship between CDAI and advanced CKM syndrome, with a potential inflection point that may help guide appropriate antioxidant intake. Although antioxidants are beneficial when consumed in appropriate amounts, our results suggest that intake beyond this threshold may not provide further benefit. Moreover, high‐dose antioxidant supplementation may carry potential risks. Excessive intake may not only disrupt normal physiological processes but also promote pro‐oxidative effects and toxicity [36, 37]. Therefore, from a practical public health perspective, we suggest that individuals aim to reach this optimal CDAI threshold primarily through a balanced diet rich in antioxidants, such as fruits, vegetables, and whole grains, rather than through high‐dose antioxidant supplementation.

Although some studies suggest potential benefits of individual dietary antioxidants in advanced CKM syndrome, others report no significant association [38]. The human body is a complex system, and interactions among dietary antioxidants are likely. Therefore, evaluating the combined effects of different antioxidants may provide deeper insight. The clinical relevance of this study lies in supporting dietary antioxidants as a potential adjunctive strategy for CKM prevention and management [38]. Clinicians may encourage individuals at risk of CKM syndrome to increase their intake of fruits and vegetables in appropriate amounts as a preventive measure [39]. Future large‐scale clinical trials are needed to clarify the specific associations between dietary nutrients and CKM progression.

This study has several strengths. First, to our knowledge, it is the first large‐scale observational study using the NHANES database to investigate the association between CDAI and CKM syndrome. This approach provides valuable insight into diet‐related multiorgan pathophysiology and strengthens the scientific relevance of the findings. Second, we adjusted for a broad range of covariates, including demographic characteristics, clinical biomarkers, and comorbid conditions. In addition, subgroup analyses were performed to assess the consistency and robustness of the results across different populations. Third, the study incorporated a mixed‐exposure model to identify key antioxidant nutrients within the CDAI, thereby improving the precision and relevance of dietary recommendations.

However, several limitations should be acknowledged. First, NHANES is a cross‐sectional survey that captures data at a single time point, which limits causal inference and does not allow assessment of temporal changes. Second, although extensive covariate adjustment was performed, residual confounding cannot be excluded and may have affected the observed associations. Third, CDAI was derived from 24‐h dietary recall data, which may not accurately reflect usual dietary intake. This method is subject to recall bias and does not capture long‐term dietary patterns. Finally, because the study population was limited to the United States, the generalizability of the findings to other regions may be limited by differences in environmental exposure, dietary habits, and nutritional status.

## 5 Conclusion

In conclusion, this study identified an L‐shaped inverse association between CDAI and advanced CKM syndrome among older adults in the United States. The findings were further supported by WQS regression, which showed a significant inverse association between combined dietary antioxidant intake and advanced CKM syndrome, with vitamin A and vitamin C emerging as the predominant contributors. These results provide new insights into the potential role of dietary antioxidants in reducing the risk of advanced CKM syndrome and offer a scientific basis for developing more targeted nutritional interventions for aging populations.

## Ethics Statement

All study protocols of NHANES were approved by the Research Ethics Review Board of the NCHS.

## Conflicts of Interest

The authors declare no conflicts of interest.

## Supporting information


**Table S1:** Definitions of CKM conditions.
**Table S2:** Detailed algorithm of the simplified 10‐year CVD risk models.
**Table S3:** Methods for evaluating each CKM stage.
**Table S4:** Distributions and concentrations of composite dietary antioxidant index (CDAI) and its components among adults in NHANES 2001–2020.
**Table S5:** Weight logistic regression analysis of quartiles of dietary antioxidant micronutrients with odds of advanced CKM syndrome among older adults in NHANES 2001–2020.
**Table S6:** Threshold effect analysis of CDAI on advanced CKM syndrome using a two‐piecewise logistic regression model in adults in the NHANES 2001–2020.
**Table S7:** Weighted logistic regression analysis of quartiles of CDAI with odds of advanced CKM syndrome after excluding extreme value of CDAI among older adults in NHANES 2001–2020 (*N* = 4934).
**Table S8:** Associations of Composite Dietary Antioxidant Index (CDAI) with CKM by subgroup.
**Figure S1:** Flowchart of study participants' selection.
**Figure S2:** Directed acyclic graph (DAG). BMI, body mass index; PIR, poverty‐to‐income ratio.
**Figure S3:** Pairwise Spearman correlation coefficients among CDAI components among older adults in NHANES 2001–2020.
**Figure S4:** Restricted cubic spline (RCS) analysis with multivariate‐adjusted associations between dietary antioxidant micronutrients and odds of advanced CKM syndrome in older adults. CDAI, composite dietary antioxidant index; CKM, Cardiovascular‐Kidney‐Metabolic. Model was adjusted for age, gender, race/ethnicity, education levels, PIR, BMI, smoking status, physical activity, alcohol intake, hypertension, and diabetes.

## Data Availability

All data relevant to the study are included in the article. The dataset was based on the NHANES (https://www.cdc.gov/nchs/nhanes/).

## References

[1] S. A. Sebastian , I. Padda , and G. Johal , “Cardiovascular‐Kidney‐Metabolic (CKM) Syndrome: A State‐Of‐The‐Art Review,” Current Problems in Cardiology 49, no. 2 (2024): 102344. 10.1016/j.cpcardiol.2023.102344 38103820

[2] C. E. Ndumele , I. J. Neeland , K. R. Tuttle , et al., “A Synopsis of the Evidence for the Science and Clinical Management of Cardiovascular‐Kidney‐Metabolic (CKM) Syndrome: A Scientific Statement From the American Heart Association,” Circulation 148, no. 20 (2023): 1636–1664. 10.1161/CIR.0000000000001186 37807920

[3] R. Aggarwal , J. W. Ostrominski , and M. Vaduganathan , “Prevalence of Cardiovascular‐Kidney‐Metabolic Syndrome Stages in US Adults, 2011–2020,” JAMA 331, no. 21 (2024): 1858–1860. 10.1001/jama.2024.6892 PMC11079779 38717747

[4] M. Marassi and G. P. Fadini , “The Cardio‐Renal‐Metabolic Connection: A Review of the Evidence,” Cardiovascular Diabetology 22, no. 1 (2023): 195. 10.1186/s12933-023-01937-x PMC10391899 37525273

[5] A. S. Go , G. M. Chertow , D. Fan , C. E. McCulloch , and C. Y. Hsu , “Chronic Kidney Disease and the Risks of Death, Cardiovascular Events, and Hospitalization,” New England Journal of Medicine 351, no. 13 (2004): 1296–1305. 10.1056/NEJMoa041031 15385656

[6] Y. L. Tain and C. N. Hsu , “Melatonin Use During Pregnancy and Lactation Complicated by Oxidative Stress: Focus on Offspring's Cardiovascular‐Kidney‐Metabolic Health in Animal Models,” Antioxidants 13, no. 2 (2024): 226. 10.3390/antiox13020226 PMC10886428 38397824

[7] L. J. Dominguez , N. Veronese , E. Baiamonte , et al., “Healthy Aging and Dietary Patterns,” Nutrients 14, no. 4 (2022): 889. 10.3390/nu14040889 PMC8879056 35215539

[8] E. Bessell , M. D. Jose , and C. McKercher , “Associations of Fish Oil and Vitamin B and E Supplementation With Cardiovascular Outcomes and Mortality in People Receiving Haemodialysis: A Review,” BMC Nephrology 16 (2015): 143. 10.1186/s12882-015-0142-1 PMC4539726 26283325

[9] D. Mozaffarian and J. H. Y. Wu , “Flavonoids, Dairy Foods, and Cardiovascular and Metabolic Health: A Review of Emerging Biologic Pathways,” Circulation Research 122, no. 2 (2018): 369–384. 10.1161/CIRCRESAHA.117.309008 PMC5781235 29348256

[10] H. Han , S. Chen , X. Wang , J. Jin , X. Li , and Z. Li , “Association of the Composite Dietary Antioxidant Index With Bone Mineral Density in the United States General Population: Data From NHANES 2005–2010,” Journal of Bone and Mineral Metabolism 41, no. 5 (2023): 631–641. 10.1007/s00774-023-01438-7 37291468

[11] M. Wang , Z. H. Huang , Y. H. Zhu , P. He , and Q. L. Fan , “Association Between the Composite Dietary Antioxidant Index and Chronic Kidney Disease: Evidence From NHANES 2011–2018,” Food and Function 14, no. 20 (2023): 9279–9286. 10.1039/d3fo01157g 37772927

[12] X. Deng , L. Ma , P. Li , et al., “Identification and Optimization of Relevant Factors for Chronic Kidney Disease in Abdominal Obesity Patients by Machine Learning Methods: Insights From NHANES 2005–2018,” Lipids in Health and Disease 23, no. 1 (2024): 390. 10.1186/s12944-024-02384-7 PMC11590401 39593076

[13] C. L. Johnson , R. Paulose‐Ram , C. L. Ogden , et al., “National Health and Nutrition Examination Survey: Analytic Guidelines, 1999–2010,” Vital and Health Statistics Series 2, Data From the National Health Survey 161 (2013): 1–24. 25090154

[14] M. E. Wright , S. T. Mayne , R. Z. Stolzenberg‐Solomon , et al., “Development of a Comprehensive Dietary Antioxidant Index and Application to Lung Cancer Risk in a Cohort of Male Smokers,” American Journal of Epidemiology 160, no. 1 (2004): 68–76. 10.1093/aje/kwh173 15229119

[15] C. E. Ndumele , J. Rangaswami , S. L. Chow , et al., “Cardiovascular‐Kidney‐Metabolic Health: A Presidential Advisory From the American Heart Association,” Circulation 148, no. 20 (2023): 1606–1635. 10.1161/CIR.0000000000001184 37807924

[16] S. S. Khan , K. Matsushita , Y. Sang , et al., “Development and Validation of the American Heart Association's PREVENT Equations,” Circulation 149, no. 6 (2024): 430–449. 10.1161/CIRCULATIONAHA.123.067626 PMC10910659 37947085

[17] C. Liu , W. Lai , M. Zhao , Y. Zhang , and Y. Hu , “Association Between the Composite Dietary Antioxidant Index and Atherosclerotic Cardiovascular Disease in Postmenopausal Women: A Cross‐Sectional Study of NHANES Data, 2013–2018,” Antioxidants 12, no. 9 (2023): 1740. 10.3390/antiox12091740 PMC10525155 37760043

[18] W. Liu , Y. Xu , L. Xiao , K. Li , and Q. Liu , “Composite Dietary Antioxidant Index Is Associated With the Prevalence of Metabolic Syndrome in Females: Results From NHANES 2011–2016,” Frontiers in Nutrition 12 (2025): 1529332. 10.3389/fnut.2025.1529332 PMC11983560 40212720

[19] L. Pan , L. Wang , H. Ma , and F. Ding , “Relevance of Combined Influence of Nutritional and Inflammatory Status on Non‐Alcoholic Fatty Liver Disease and Advanced Fibrosis: A Mediation Analysis of Lipid Biomarkers,” Journal of Gastroenterology and Hepatology 39, no. 12 (2024): 2853–2862. 10.1111/jgh.16760 39392197

[20] M. H. Wei , Y. Cui , H. L. Zhou , et al., “Associations of Multiple Metals With Bone Mineral Density: A Population‐Based Study in US Adults,” Chemosphere 282 (2021): 131150. 10.1016/j.chemosphere.2021.131150 34470175

[21] R. Zhu , R. Wang , J. He , et al., “Prevalence of Cardiovascular‐Kidney‐Metabolic Syndrome Stages by Social Determinants of Health,” JAMA Network Open 7, no. 11 (2024): e2445309. 10.1001/jamanetworkopen.2024.45309 PMC11574692 39556396

[22] B. Dong , Y. Chen , X. Yang , et al., “Estimated Glucose Disposal Rate Outperforms Other Insulin Resistance Surrogates in Predicting Incident Cardiovascular Diseases in Cardiovascular‐Kidney‐Metabolic Syndrome Stages 0–3 and the Development of a Machine Learning Prediction Model: A Nationwide Prospective Cohort Study,” Cardiovascular Diabetology 24, no. 1 (2025): 163. 10.1186/s12933-025-02729-1 PMC12004813 40241176

[23] R. Ma , X. Zhou , G. Zhang , et al., “Association Between Composite Dietary Antioxidant Index and Coronary Heart Disease Among US Adults: A Cross‐Sectional Analysis,” BMC Public Health 23, no. 1 (2023): 2426. 10.1186/s12889-023-17373-1 PMC10699074 38053099

[24] M. Zhao , D. Zhang , Q. Zhang , Y. Lin , and H. Cao , “Association Between Composite Dietary Antioxidant Index and Hyperlipidemia: A Cross‐Sectional Study From NHANES (2005–2020),” Scientific Reports 14, no. 1 (2024): 15935. 10.1038/s41598-024-66922-0 PMC11237065 38987566

[25] Z. Wang , Q. Wang , F. Tang , and S. Zhong , “Composite Dietary Antioxidant Index and Obesity Among U.S. Adults in NHANES 2007–2018,” Scientific Reports 14, no. 1 (2024): 28102. 10.1038/s41598-024-78852-y PMC11564541 39543203

[26] X. Hu , Z. Zhao , Q. An , Y. Li , and B. Wang , “Association of Independent Dietary Antioxidant Intake, and CDAI Level With Risks of All‐Cause and Cardiovascular‐Cause Death Among Population With Cardiovascular Disease,” BMC Public Health 25, no. 1 (2025): 1327. 10.1186/s12889-025-22481-1 PMC11980126 40205537

[27] M. J. Haskell , “The Challenge to Reach Nutritional Adequacy for Vitamin A: β‐Carotene Bioavailability and Conversion—Evidence in Humans,” American Journal of Clinical Nutrition 96, no. 5 (2012): 1193s–1203s. 10.3945/ajcn.112.034850 23053560

[28] G. Tang , “Bioconversion of Dietary Provitamin A Carotenoids to Vitamin A in Humans,” American Journal of Clinical Nutrition 91, no. 5 (2010): 1468s–1473s. 10.3945/ajcn.2010.28674G PMC2854912 20200262

[29] J. von Lintig , “Provitamin A Metabolism and Functions in Mammalian Biology,” American Journal of Clinical Nutrition 96, no. 5 (2012): 1234s–1244s. 10.3945/ajcn.112.034629 PMC3471205 23053549

[30] S. Fu , L. Zhang , F. Ma , S. Xue , T. Sun , and Z. Xu , “Effects of Selenium on Chronic Kidney Disease: A Mendelian Randomization Study,” Nutrients 14, no. 21 (2022): 4458. 10.3390/nu14214458 PMC9654848 36364721

[31] Y. Pi , X. Liao , X. Song , et al., “Association Between Dietary Intake of Selenium and Chronic Kidney Disease in US Adults: A Cross‐Sectional Study of NHANES 2015–2018,” Frontiers in Nutrition 11 (2024): 1396470. 10.3389/fnut.2024.1396470 PMC11347418 39193560

[32] C. Xie , M. Zeng , Z. Shi , S. Li , K. Jiang , and Y. Zhao , “Association Between Selenium Status and Chronic Kidney Disease in Middle‐Aged and Older Chinese Based on CHNS Data,” Nutrients 14, no. 13 (2022): 2695. 10.3390/nu14132695 PMC9269073 35807874

[33] Y. H. Wei , C. Y. Lu , C. Y. Wei , Y. S. Ma , and H. C. Lee , “Oxidative Stress in Human Aging and Mitochondrial Disease‐Consequences of Defective Mitochondrial Respiration and Impaired Antioxidant Enzyme System,” Chinese Journal of Physiology 44, no. 1 (2001): 1–11. 11403514

[34] M. J. Tavassolifar , M. Changaei , Z. Salehi , et al., “Redox Imbalance in Crohn's Disease Patients Is Modulated by Azathioprine,” Redox Report 26, no. 1 (2021): 80–84. 10.1080/13510002.2021.1915665 PMC8079067 33882797

[35] A. M. Pisoschi and A. Pop , “The Role of Antioxidants in the Chemistry of Oxidative Stress: A Review,” European Journal of Medicinal Chemistry 97 (2015): 55–74. 10.1016/j.ejmech.2015.04.040 25942353

[36] J. Mao , Y. Zhao , H. Hu , M. Zhou , and X. Yang , “An L‐Shaped Association Between Composite Dietary Antioxidant Index and Stroke: Evidence From NHANES 2011–2020,” Journal of Stroke and Cerebrovascular Diseases 33, no. 3 (2024): 107578. 10.1016/j.jstrokecerebrovasdis.2024.107578 38232583

[37] Q. H. Tan , Y. Q. Huang , X. C. Liu , et al., “A U‐Shaped Relationship Between Selenium Concentrations and All‐Cause or Cardiovascular Mortality in Patients With Hypertension,” Frontiers in Cardiovascular Medicine 8 (2021): 671618. 10.3389/fcvm.2021.671618 PMC8360873 34395551

[38] K. C. Ferdinand , “An Overview of Cardiovascular‐Kidney‐Metabolic Syndrome,” American Journal of Managed Care 30, no. 10 (2024): S181–s188. 10.37765/ajmc.2024.89670 39705194

[39] S. L. Hung , T. Y. Lin , and S. C. Hung , “Adherence to a Healthy Plant‐Based Diet and Cardiovascular‐Kidney‐Metabolic Risk Factors in Patients With Moderate to Advanced Chronic Kidney Disease,” Journal of the American Nutrition Association 44, no. 7 (2025): 651–660. 10.1080/27697061.2025.2488366 40277953

