# Supplementary material for: Joint and Independent Associations of Dietary Antioxidant Intakes With Advanced Stages in Older Patients With Cardiovascular‐Kidney‐Metabolic Syndrome
Source: Kaohsiung J Med Sci. 2026 May 13:e70215. Online ahead of print. doi: 10.1002/kjm2.70215 (PMC13399601; doi:10.1002/kjm2.70215)
Supplement: Supplementary file 1 — Table S1: Definitions of CKM conditions. Table S2: Detailed algorithm of the simplified 10‐year CVD risk models. Table S3: Methods for evaluating each CKM stage. Table S4: Distributions and concentrations of composite dietary antioxidant index (CDAI) and its components among adults in NHANES 2001–2020. Table S5: Weight logistic regression analysis of quartiles of dietary antioxidant micronutrients with odds of advanced CKM syndrome among older adults in NHANES 2001–2020. Table S6: Threshold effect analysis of CDAI on advanced CKM syndrome using a two‐piecewise logistic regression model in adults in the NHANES 2001–2020. Table S7: Weighted logistic regression analysis of quartiles of CDAI with odds of advanced CKM syndrome after excluding extreme value of CDAI among older adults in NHANES 2001–2020 (N = 4934). Table S8: Associations of Composite Dietary Antioxidant Index (CDAI) with CKM by subgroup. Figure S1: Flowchart of study participants' selection. Figure S2: Directed acyclic graph (DAG). BMI, body mass index; PIR, poverty‐to‐income ratio. Figure S3: Pairwise Spearman correlation coefficients among CDAI components among older adults in NHANES 2001–2020. Figure S4: Restricted cubic spline (RCS) analysis with multivariate‐adjusted associations between dietary antioxidant micronutrients and odds of advanced CKM syndrome in older adults. CDAI, composite dietary antioxidant index; CKM, Cardiovascular‐Kidney‐Metabolic. Model was adjusted for age, gender, race/ethnicity, education levels, PIR, BMI, smoking status, physical activity, alcohol intake, hypertension, and diabetes. [file KJM2-9999-e70215-s001.docx]

**Supplementary Materials**

**Table S1** Definitions of CKM conditions.

**Table S1** Detailed algorithm of the simplified 10-year CVD risk models.

**Table S3** Methods for evaluating each CKM stage.

**Table S4** Distributions and concentrations of composite dietary antioxidant index (CDAI) and its components among adults in NHANES 2001–2020.

**Table S5** Weight logistic regression analysis of quartiles of dietary antioxidant micronutrients with odds of advanced CKM syndrome among older adults in NHANES 2001–2020.

**Table S6** Threshold effect analysis of CDAI on advanced CKM syndrome using a two-piecewise logistic regression model in adults in the NHANES 2001-2020.

**Table S7** Weighted logistic regression analysis of quartiles of CDAI with odds of advanced CKM syndrome after excluding extreme value of CDAI among older adults in NHANES 2001 to 2020 (N=4934).

**Table S8** Associations of Composite Dietary Antioxidant Index (CDAI) with CKM by Subgroup.

**Figure S1** Flowchart of study participants' selection.

**Figure S2** Directed acyclic graph (DAG). Abbreviations: *BMI*, body mass index; *PIR*, poverty-to-income ratio.

**Figure S3** Pairwise Spearman correlation coefficients among CDAI components among older adults in NHANES 2001 to 2020.

**Figure S4** Restricted cubic spline (RCS) analysis with multivariate-adjusted associations between dietary antioxidant micronutrients and odds of advanced CKM syndrome in older adults. Abbreviations: *CDAI*, composite dietary antioxidant index; *CKM*, Cardiovascular-Kidney-Metabolic. Model was adjusted for age, gender, race/ethnicity, education levels, PIR, BMI, smoking status, physical activity, alcohol intake, hypertension, and diabetes.

**Table S1** Definitions of CKM conditions.

| CKM  conditions | Definition | CKM indicators | Threshold for CKM indicators |
| --- | --- | --- | --- |
| CVD | Individuals with clinical CVD or subclinical CVD | Clinical CVD | History of chronic heart failure, coronary heart disease, heart attack, or stroke |
|  |  | Subclinical CVD | Any of the following criterion is met:  1) Very high-risk CKD in KDIGO classification: UACR ≥ 300 mg/g and eGFR ≤ 45-59 ml/min/1.73m2 , UACR ≥ 30 mg/g and eGFR ≤ 30-44 ml/min/1.73m2 , or eGFR ≤ 29 ml/min/1.73m2 .  2) Predicted 10-year CVD risk ≥ 20% |
| Kidney  diseases | Individuals with CKD | CKD | Moderate-to-high-risk CKD in KDIGO classification: UACR ≥ 30 mg/g and eGFR ≥ 60 ml/min/1.73m2 , UACR < 300 mg/g and eGFR ≤ 45-59 ml/min/1.73m2 , or UACR < 30 mg/g and eGFR ≤ 30-44 ml/min/1.73m2 . |
| Metabolic  disorders | Individuals with overweight/obesity, abdominal obesity, prediabetes,  diabetes, hypertension,  hypertriglyceridemia or MetS | Overweight/obesity | BMI ≥25 kg/m2 (or ≥23 kg/m2 if Asian ancestry) * |
|  |  | Abdominal obesity | Waist circumference ≥88/102 cm in female/male (or if Asian ancestry ≥80/90 cm in female/male) |
|  |  | Prediabetes | Fasting blood glucose ≥ 100-124 mg/dL or HbA1c ≥ 5.7%-6.4% and without self-reported diagnosis of diabetes, use of insulin, or oral  hypoglycemic agents |
|  |  | Diabetes | Fasting blood glucose ≥ 125 mg/dL or HbA1c ≥ 6.5% or self-reported diagnosis of diabetes, use of insulin, or oral hypoglycemic agents |
|  |  | Hypertension | SBP ≥130 mm Hg or DBP ≥80 mm Hg or self-reported diagnosis of hypertension or use of antihypertensive medications |
|  |  | Hypertriglyceridemia | Triglycerides ≥ 135 mg/dL |
|  |  | MetS | MetS is defined by the presence of 3 or more of the following: |

|  |  |  | 1) Waist circumference ≥88/102 cm in female/male (or if Asian ancestry ≥80/90 cm in female/male).  2) HDL cholesterol ≥50/40 mg/dL in female/male.  3) Triglycerides ≥150 mg/dL.  4) Elevated blood pressure (SBP ≥130 mm Hg or DBP ≥80 mm Hg and/or use of antihypertensive medications)  5) Fasting blood glucose ≥100 mg/dL |
| --- | --- | --- | --- |

**Abbreviations:** BMI: body mass index; CKD: chronic kidney disease; CKM: cardiovascular-kidney-metabolic; CVD: cardiovascular disease; DBP: diastolic

blood pressure; eGFR: estimated glomerular filtration rate; HDL: high-density lipoprotein; KDIGO: The Kidney Disease: Improving Global Outcomes; MetS:

metabolic syndrome; SBP: systolic blood pressure; UACR: urinary albumin to creatinine ratio.

* Asian was not listed as a separate race/ethnicity until NAHNES 2011-2012, therefore the uniform threshold for BMI and waist circumference was used in all participants in NHANES 1999-2010.

**Table S2** Detailed algorithm of the simplified 10-year CVD risk models.

| Women | **log-Odds** = -3.307728 + 0.7939329 × (age – 55) /10 +  0.0305239 × (TC – HDL-C – 3.5) – 0.1606857 × (HDL-C –  1.3) /0.3 – 0.2394003 × (min(SBP, 110) – 110) /20 + 0.360078 × (max(SBP, 110) – 130) /20 + 0.8667604 × (if diabetes) +  0.5360739 × (if current smoker) + 0.6045917 × (min(eGFR, 60) – 60) / -15 + 0.0433769 × (max(eGFR, 60) – 90) / -15 + 0.3151672 × (if using anti-hypertensive medication) –  0.1477655 × (if using statin) – 0.0663612 × (if using anti-  hypertensive medication) × (max(SBP, 110) – 130) /20 +  0.1197879 × (if using statin) × (TC – HDL-C – 3.5) –  0.0819715 × (age – 55) /10 × (TC – HDL-C – 3.5) +  0.0306769 × (age – 55) /10 × (HDL-C – 1.3) /0.3 – 0.0946348 × (age – 55) /10 × (max(SBP, 110) – 130) /20 – 0.27057 ×  (age – 55) /10 × (if diabetes) – 0.078715 × (age – 55) /10 × (if current smoker) – 0.1637806 × (age – 55) /10 × (min(eGFR, 60) – 60) / -15  **Risk** = exp(log-Odds) / (1 + exp(log-Odds)) |
| --- | --- |
| Men | **log-Odds** = -3.031168 + 0.7688528 × (age – 55) /10 +  0.0736174 × (TC – HDL-C – 3.5) – 0.0954431 × (HDL-C –  1.3) /0.3 – 0.4347345 × (min(SBP, 110) – 110) /20 +  0.3362658 × (max(SBP, 110) – 130) /20 + 0.7692857 × (if  diabetes) + 0.4386871 × (if current smoker) + 0.5378979 × (min(eGFR, 60) – 60) / -15 + 0.0164827 × (max(eGFR, 60) – 90) / -15 + 0.288879 × (if using anti-hypertensive medication)  – 0.1337349 × (if using statin) – 0.0475924 × (if using anti-  hypertensive medication) × (max(SBP, 110) – 130) /20 +  0.150273 × (if using statin) × (TC – HDL-C – 3.5) – 0.0517874 × (age – 55) /10 × (TC – HDL-C – 3.5) + 0.0191169 × (age – 55) /10 × (HDL-C – 1.3) /0.3 – 0.1049477 × (age – 55) /10 × (max(SBP, 110) – 130) /20 – 0.2251948 × (age – 55) /10 × (if diabetes) – 0.0895067 × (age – 55) /10 × (if current smoker) – 0.1543702 × (age – 55) /10 × (min(eGFR, 60) – 60) / -15  **Risk** = exp(log-Odds) / (1 + exp(log-Odds)) |

**Abbreviations**: eGFR: estimated glomerular filtration rate; HDL: high-density lipoprotein cholesterol; SBP: systolic blood pressure; TC: total cholesterol.

**Table S3** Methods for evaluating each CKM stage.

| CKM stages | Definition | Criterion | Threshold for CKM conditions |
| --- | --- | --- | --- |
| Stage 0: No  CKM risk factors | Individuals with normal BMI and waist circumference, normoglycemia,  normotension, a normal lipid profile, and no evidence of CKD or subclinical or clinical CVD | All criteria are met | BMI <25 kg/m2 (or <23 kg/m2 if Asian ancestry) * |
|  |  |  | Waist circumference <88/102 cm in female/male (or if Asian ancestry <80/90 cm in female/male) |
|  |  |  | Fasting blood glucose < 100 mg/dL and HbA1c < 5.7% and without self-reported diagnosis of diabetes, use of insulin, or oral  hypoglycemic agents |
|  |  |  | SBP <130 mm Hg and DBP <80 mm Hg without self-reported  diagnosis of hypertension or use of antihypertensive medications |
|  |  |  | HDL cholesterol <50/40 mg/dL in female/male and triglycerides < 150 mg/dL |
|  |  |  | Low-risk CKD in KDIGO classification according to eGFR and UACR: UACR < 30 mg/g and eGFR ≥ 60 ml/min/1.73m2 . |
|  |  |  | Predicted 10-year CVD risk < 20% |
|  |  |  | No clinical CVD |
| Stage 1: Excess or dysfunctional adiposity | Individuals with overweight/obesity,  abdominal obesity, or dysfunctional  adipose tissue, without the presence of other metabolic risk factors or CKD | Any of the three criteria is met | Overweight/obesity |
|  |  |  | Abdominal obesity |
|  |  |  | Prediabetes |
|  |  | All criteria are met | SBP <130 mm Hg and DBP <80 mm Hg without self-reported  diagnosis of hypertension or use of antihypertensive medications |
|  |  |  | HDL cholesterol <50/40 mg/dL in female/male and triglycerides < 150 mg/dL |
|  |  |  | Low-risk CKD in KDIGO classification according to eGFR and UACR: UACR < 30 mg/g and eGFR ≥ 60 ml/min/1.73m2 . |

|  |  |  | Predicted 10-year CVD risk < 20% |
| --- | --- | --- | --- |
|  |  |  | No clinical CVD |
| Stage 2:  Metabolic risk  factors and CKD | Individuals with metabolic risk factors (hypertriglyceridemia, hypertension, MetS, diabetes), or CKD | Any of the five criteria is met | Hypertriglyceridemia |
|  |  |  | Hypertension |
|  |  |  | diabetes |
|  |  |  | MetS |
|  |  |  | Moderate-to-high-risk CKD in KDIGO classification |
|  |  | All criteria are met | No very high-risk CKD in KDIGO classification |
|  |  |  | Predicted 10-year CVD risk < 20% |
|  |  |  | No clinical CVD |
| Stage 3:  Subclinical CVD in CKM | Subclinical CVD among individuals with excess/dysfunctional adiposity, other metabolic risk factors, or CKD | Any of the two criteria is met | Very high-risk CKD in KDIGO classification |
|  |  |  | Predicted 10-year CVD risk ≥ 20% |
|  |  | Any of the eight criteria is met | Overweight/obesity |
|  |  |  | Abdominal obesity |
|  |  |  | Prediabetes |
|  |  |  | Hypertriglyceridemia |
|  |  |  | Hypertension |
|  |  |  | diabetes |
|  |  |  | MetS |
|  |  |  | Moderate-to-high-risk CKD in KDIGO classification |
|  |  | The criterion is met | No clinical CVD |
| Stage 4: Clinical CVD in CKM | Clinical CVD among individuals with excess/dysfunctional adiposity,  other metabolic risk factors, or CKD | The criterion is met | Clinical CVD |
|  |  | Any of the nine criteria is met | Overweight/obesity |
|  |  |  | Abdominal obesity |
|  |  |  | Prediabetes |

|  |  |  | Hypertriglyceridemia |
| --- | --- | --- | --- |
|  |  |  | Hypertension |
|  |  |  | diabetes |
|  |  |  | MetS |
|  |  |  | Moderate-to-high-risk CKD in KDIGO classification |
|  |  |  | Very high-risk CKD in KDIGO classification |

**Abbreviations:** BMI: body mass index; CKD: chronic kidney disease; CKM: cardiovascular-kidney-metabolic; CVD: cardiovascular disease; DBP: diastolic

blood pressure; eGFR: estimated glomerular filtration rate; HDL: high-density lipoprotein; KDIGO: The Kidney Disease: Improving Global Outcomes; SBP:

systolic blood pressure; UACR: urinary albumin to creatinine ratio.

* Asian was not listed as a separate race/ethnicity until NAHNES 2011-2012, therefore the uniform threshold for BMI and waist circumference was used in all participants in NHANES 1999-2010.

**Table S4** Distributions and concentrations of composite dietary antioxidant index (CDAI) and its components among adults in NHANES 2001–2020.

|  | Mean | 5^th^ | 25^th^ | 50^th^ | 75^th^ | 95^th^ |
| --- | --- | --- | --- | --- | --- | --- |
| CDAI | 0.18 | -4.59 | -2.33 | -0.42 | 1.96 | 6.77 |
| Vitamins A, μg/day | 636.70 | 140.83 | 335.25 | 537.00 | 786.50 | 1396.68 |
| Vitamins C, mg/day | 85.43 | 9.70 | 32.91 | 66.95 | 116.09 | 223.80 |
| Vitamins E, mg/day | 7.23 | 2.31 | 4.25 | 6.20 | 8.74 | 15.89 |
| Zinc, mg/day | 2.21 | 1.39 | 1.91 | 2.22 | 2.53 | 3.00 |
| Selenium, μg/day | 99.66 | 41.98 | 68.05 | 92.45 | 121.55 | 180.64 |
| Carotenoid, μg/day | 9192.71 | 696.33 | 2859.13 | 6279.25 | 12436.38 | 26866.60 |

N, number of urinary samples; 5^th^, 5th percentile; 25^th^, 25th percentile; 50^th^, 50th percentile; 75^th^, 75th percentile; 95^th^, 95th percentile.

**Table S5** Weight logistic regression analysis of quartiles of dietary antioxidant micronutrients with odds of advanced CKM syndrome among older adults in NHANES 2001–2020.

|  | Crude | |  | Model 1 | |  | Model 2 | |
| --- | --- | --- | --- | --- | --- | --- | --- | --- |
|  | β (95% CI) | *P* value |  | β (95% CI) | *P* value |  | β (95% CI) | *P* value |
| Quartiles of Vitamins A | |  |  |  |  |  |  |  |
| Quartile 1 | 0 [Reference] |  |  | 0 [Reference] |  |  | 0 [Reference] |  |
| Quartile 2 | 1.01 (0.86, 1.19) | 0.879 |  | 0.85 (0.71, 1.02) | 0.085 |  | 0.81 (0.67, 0.99) | 0.042 |
| Quartile 3 | 0.95 (0.81, 1.11) | 0.517 |  | 0.71 (0.59, 0.85) | <0.001 |  | 0.71 (0.58, 0.87) | 0.001 |
| Quartile 4 | 1.01 (0.86, 1.18) | 0.932 |  | 0.73 (0.61, 0.88) | 0.001 |  | 0.73 (0.60, 0.89) | 0.002 |
| *P* for trend |  | 0.864 |  |  | <0.001 |  |  | 0.001 |
| Quartiles of Vitamins C | |  |  |  |  |  |  |  |
| Quartile 1 | 0 [Reference] |  |  | 0 [Reference] |  |  | 0 [Reference] |  |
| Quartile 2 | 0.79 (0.67, 0.92) | 0.003 |  | 0.67 (0.56, 0.80) | <0.001 |  | 0.69 (0.57, 0.84) | <0.001 |
| Quartile 3 | 0.78 (0.66, 0.91) | 0.002 |  | 0.65 (0.54, 0.78) | <0.001 |  | 0.72 (0.59, 0.87) | 0.001 |
| Quartile 4 | 0.74 (0.63, 0.87) | <0.001 |  | 0.61 (0.50, 0.73) | <0.001 |  | 0.74 (0.60, 0.90) | 0.003 |
| *P* for trend |  | <0.001 |  |  | <0.001 |  |  | 0.006 |
| Quartiles of Vitamins E | |  |  |  |  |  |  |  |
| Quartile 1 | 0 [Reference] |  |  | 0 [Reference] |  |  | 0 [Reference] |  |
| Quartile 2 | 0.95 (0.81, 1.12) | 0.547 |  | 0.98 (0.82, 1.18) | 0.863 |  | 1.00 (0.82, 1.22) | 0.978 |
| Quartile 3 | 0.80 (0.68, 0.93) | 0.005 |  | 0.86 (0.71, 1.02) | 0.089 |  | 0.84 (0.68, 1.02) | 0.080 |
| Quartile 4 | 0.71 (0.60, 0.83) | <0.001 |  | 0.83 (0.69, 1.00) | 0.046 |  | 0.88 (0.72, 1.08) | 0.230 |
| *P* for trend |  | <0.001 |  |  | 0.018 |  |  | 0.089 |
| Quartiles of Zinc | |  |  |  |  |  |  |  |
| Quartile 1 | 0 [Reference] |  |  | 0 [Reference] |  |  | 0 [Reference] |  |
| Quartile 2 | 0.86 (0.73, 1.01) | 0.062 |  | 0.95 (0.79, 1.13) | 0.560 |  | 0.87 (0.72, 1.06) | 0.180 |
| Quartile 3 | 0.81 (0.70, 0.95) | 0.011 |  | 0.92 (0.76, 1.10) | 0.345 |  | 0.79 (0.65, 0.97) | 0.024 |
| Quartile 4 | 0.88 (0.75, 1.03) | 0.124 |  | 1.08 (0.90, 1.30) | 0.398 |  | 0.86 (0.69, 1.05) | 0.144 |
| *P* for trend |  | 0.095 |  |  | 0.494 |  |  | 0.097 |
| Quartiles of Selenium | |  |  |  |  |  |  |  |
| Quartile 1 | 0 [Reference] |  |  | 0 [Reference] |  |  | 0 [Reference] |  |
| Quartile 2 | 0.90 (0.77, 1.05) | 0.179 |  | 0.97 (0.81, 1.16) | 0.754 |  | 0.92 (0.75, 1.12) | 0.390 |
| Quartile 3 | 0.85 (0.73, 1.00) | 0.049 |  | 1.02 (0.85, 1.22) | 0.864 |  | 0.89 (0.73, 1.09) | 0.259 |
| Quartile 4 | 0.70 (0.60, 0.82) | <0.001 |  | 1.02 (0.85, 1.22) | 0.824 |  | 0.74 (0.60, 0.92) | 0.006 |
| *P* for trend |  | <0.001 |  |  | 0.718 |  |  | 0.007 |
| Quartiles of Carotenoid | |  |  |  |  |  |  |  |
| Quartile 1 | 0 [Reference] |  |  | 0 [Reference] |  |  | 0 [Reference] |  |
| Quartile 2 | 0.85 (0.73, 1.00) | 0.047 |  | 0.83 (0.69, 0.99) | 0.037 |  | 0.91 (0.75, 1.10) | 0.335 |
| Quartile 3 | 0.70 (0.59, 0.82) | <0.001 |  | 0.67 (0.56, 0.80) | <0.001 |  | 0.76 (0.63, 0.93) | 0.008 |
| Quartile 4 | 0.71 (0.60, 0.83) | <0.001 |  | 0.69 (0.58, 0.83) | <0.001 |  | 0.81 (0.67, 0.99) | 0.039 |
| *P* for trend |  | <0.001 |  |  | <0.001 |  |  | 0.013 |

Model 1 was adjusted for age, gender, race/ethnicity; Model 2 was adjusted as model 1 plus education levels, PIR, BMI, smoking status, physical activity, alcohol intake, hypertension, and diabetes.

**Table S6** Threshold effect analysis of CDAI on advanced CKM syndrome using a two-piecewise logistic regression model in adults in the NHANES 2001-2020.

| Threshold effect analysis | Advanced CKM syndrome | |
| --- | --- | --- |
|  | OR (95% CI) | *P*-value |
| Inflection point of CDAI (K) | 5.857 | |
| <K slope | 0.951 (0.925, 0.978) | <0.001 |
| >K slope | 1.070 (0.992, 1.154) | 0.204 |
| Log-likelihood ratio test | 0.001 | |

Abbreviations: CDAI, composite dietary antioxidant index; *CKM*, Cardiovascular-Kidney-Metabolic; *OR*, odds ratio; *CI*, confidence interval; *NHANES*, National Health and Nutrition Examination Survey.

Model was adjusted for age, gender, race/ethnicity, education levels, PIR, BMI, smoking status, physical activity, alcohol intake, hypertension, and diabetes.

**Table S7** Weighted logistic regression analysis of quartiles of CDAI with odds of advanced CKM syndrome after excluding extreme value of CDAI among older adults in NHANES 2001 to 2020 (N=4934).

|  | Crude | | Model 1 | | Model 2 | |
| --- | --- | --- | --- | --- | --- | --- |
|  | OR (95% CI) | P-value | OR (95% CI) | P-value | OR (95% CI) | P-value |
| Per 1 unit increase | 0.943 (0.926, 0.959) | <0.001 | 0.945 (0.927, 0.964) | <0.001 | 0.972 (0.951, 0.993) | 0.010 |
| Quartiles of CDAI |  |  |  |  |  |  |
| Quartile 1 | Reference |  |  |  | Reference |  |
| Quartile 2 | 0.788 (0.672, 0.923) | 0.003 | 0.773 (0.646, 0.924) | 0.005 | 0.849 (0.698, 1.033) | 0.101 |
| Quartile 3 | 0.690 (0.589, 0.809) | <0.001 | 0.684 (0.571, 0.820) | <0.001 | 0.776 (0.635, 0.948) | 0.013 |
| Quartile 4 | 0.558 (0.475, 0.656) | <0.001 | 0.561 (0.467, 0.674) | <0.001 | 0.729 (0.594, 0.895) | 0.003 |
| P for trend | <0.001 | | <0.001 | | 0.002 | |

Abbreviations: *CDAI*, composite dietary antioxidant index; *CKM*, Cardiovascular-Kidney-Metabolic; *OR*, odds ratio; *CI*, confidence interval; *NHANES*, National Health and Nutrition Examination Survey.

Model 1 was adjusted for age, gender, race/ethnicity; Model 2 was adjusted as model 1 plus education levels, PIR, BMI, smoking status, physical activity, alcohol intake, hypertension, and diabetes.

**Table S8** Associations of Composite Dietary Antioxidant Index (CDAI) with CKM by Subgroup.

| **Characteristic** | **OR (95%CI)** | ***P*-value** | ***P* for interaction** |
| --- | --- | --- | --- |
| **Age (%)** |  |  | 0.897 |
| ≤70 | 0.95 (0.93, 0.97) | <0.001 |  |
| ＞70 | 0.95 (0.93, 0.97) | <0.001 |  |
| **Gender (%)** |  |  | 0.331 |
| Male | 0.94 (0.92, 0.96) | <0.001 |  |
| Female | 0.96 (0.93, 0.98) | <0.001 |  |
| **BMI (%)** |  |  | 0.763 |
| Normal<25 | 0.94 (0.91, 0.97) | <0.001 |  |
| Overweight 25–30 | 0.95 (0.93, 0.98) | <0.001 |  |
| Obese>30 | 0.95 (0.89, 0.97) | <0.001 |  |
| **Race (%)** |  |  | 0.509 |
| Non-Hispanic white | 0.95 (0.93, 0.97) | <0.001 |  |
| Non-Hispanic black | 0.95 (0.91, 0.98) | 0.003 |  |
| Mexican American | 0.91 (0.87, 0.96) | <0.001 |  |
| Others | 0.93 (0.88, 0.98) | 0.005 |  |
| **PIR (%)** |  |  | 0.234 |
| 0.00–1.30 | 0.96 (0.93, 0.99) | 0.021 |  |
| 1.31–3.50 | 0.97 (0.95, 0.99) | 0.015 |  |
| >3.50 | 0.94 (0.91, 0.97) | <0.001 |  |
| **Education (%)** |  |  | 0.557 |
| Less than High school or equivalent | 0.96 (0.93, 0.99) | 0.009 |  |
| High school or equivalent | 0.97 (0.94, 1.00) | 0.08 |  |
| College or above | 0.95 (0.93, 0.97) | <0.001 |  |
| **Smoking status (%)** |  |  | 0.082 |
| Non-smoking | 0.96 (0.94, 0.98) | 0.001 |  |
| Passive smoking | 0.93 (0.91, 0.96) | <0.0001 |  |
| Active smoking | 0.98 (0.94, 1.02) | 0.392 |  |
| **Alcohol drinking (%)** |  |  | 0.459 |
| Non-drinking | 0.95 (0.91, 0.99) | 0.013 |  |
| Low to moderate drinking | 0.94 (0.91, 0.97) | <0.001 |  |
| Heavy drinking | 0.96 (0.94, 0.98) | 0.001 |  |
| **Physical activity (%)** |  |  | 0.304 |
| No | 0.95 (0.93, 0.97) | <0.001 |  |
| Yes | 0.96 (0.94, 0.99) | 0.001 |  |
| **Hypertension (%)** |  |  | 0.963 |
| No | 0.95 (0.91, 0.99) | 0.013 |  |
| Yes | 0.95 (0.93, 0.97) | <0.001 |  |
| **Diabetes (%)** |  |  | 0.058 |
| No | 0.97 (0.95, 0.98) | 0.001 |  |
| Yes | 0.93 (0.91, 0.96) | <0.001 |  |

Abbreviations: *CKM*, Cardiovascular-Kidney-Metabolic; *BMI*, body mass index; *PIR*, poverty-to-income ratio; *OR*, odds ratio; *CI*, confidence interval.

Multivariable-adjusted model was adjusted for age, gender, race/ethnicity, education levels, PIR, BMI, smoking status, physical activity, alcohol intake, hypertension, and diabetes.


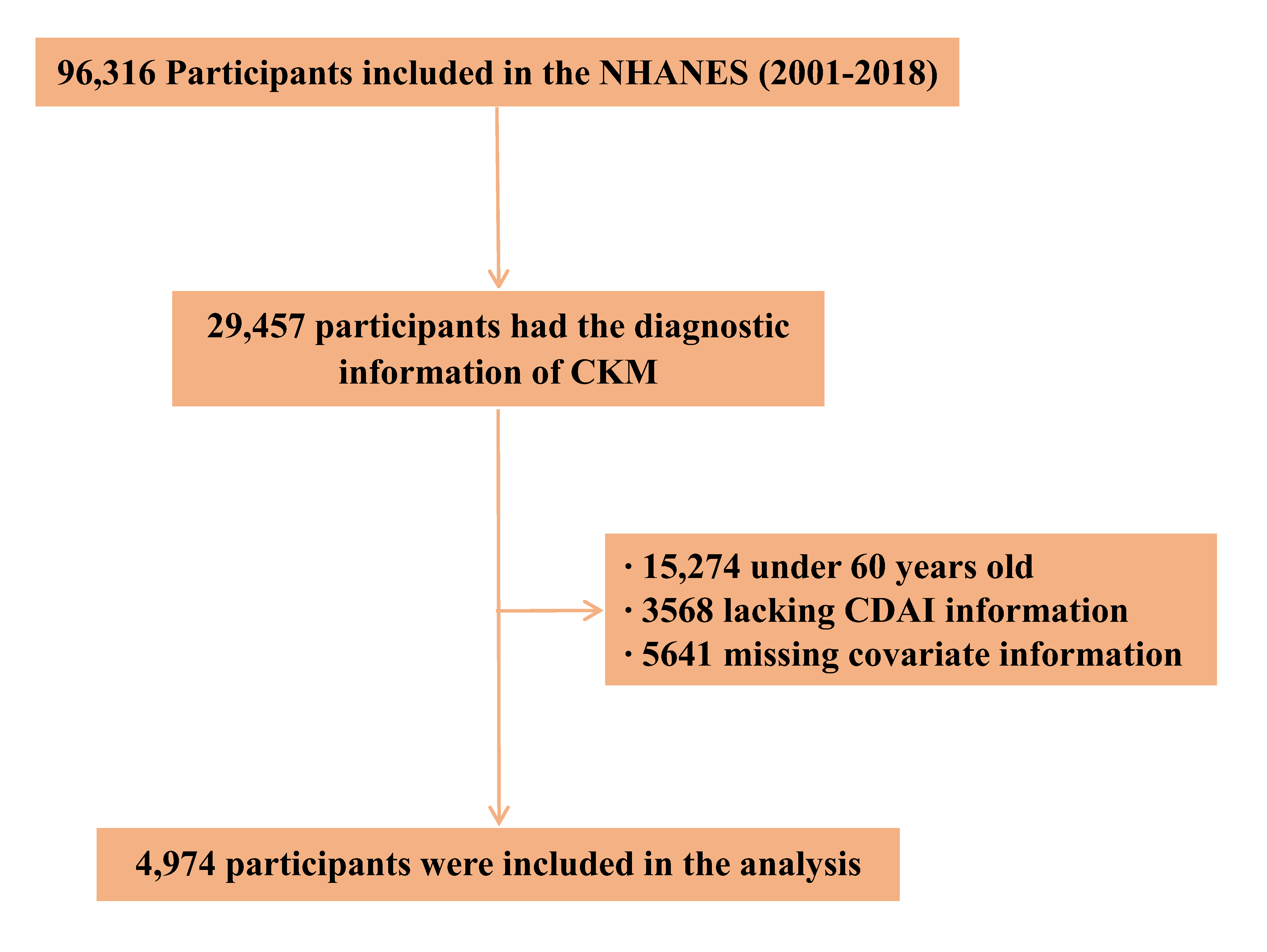


**Figure S1** Flowchart of study participants' selection.


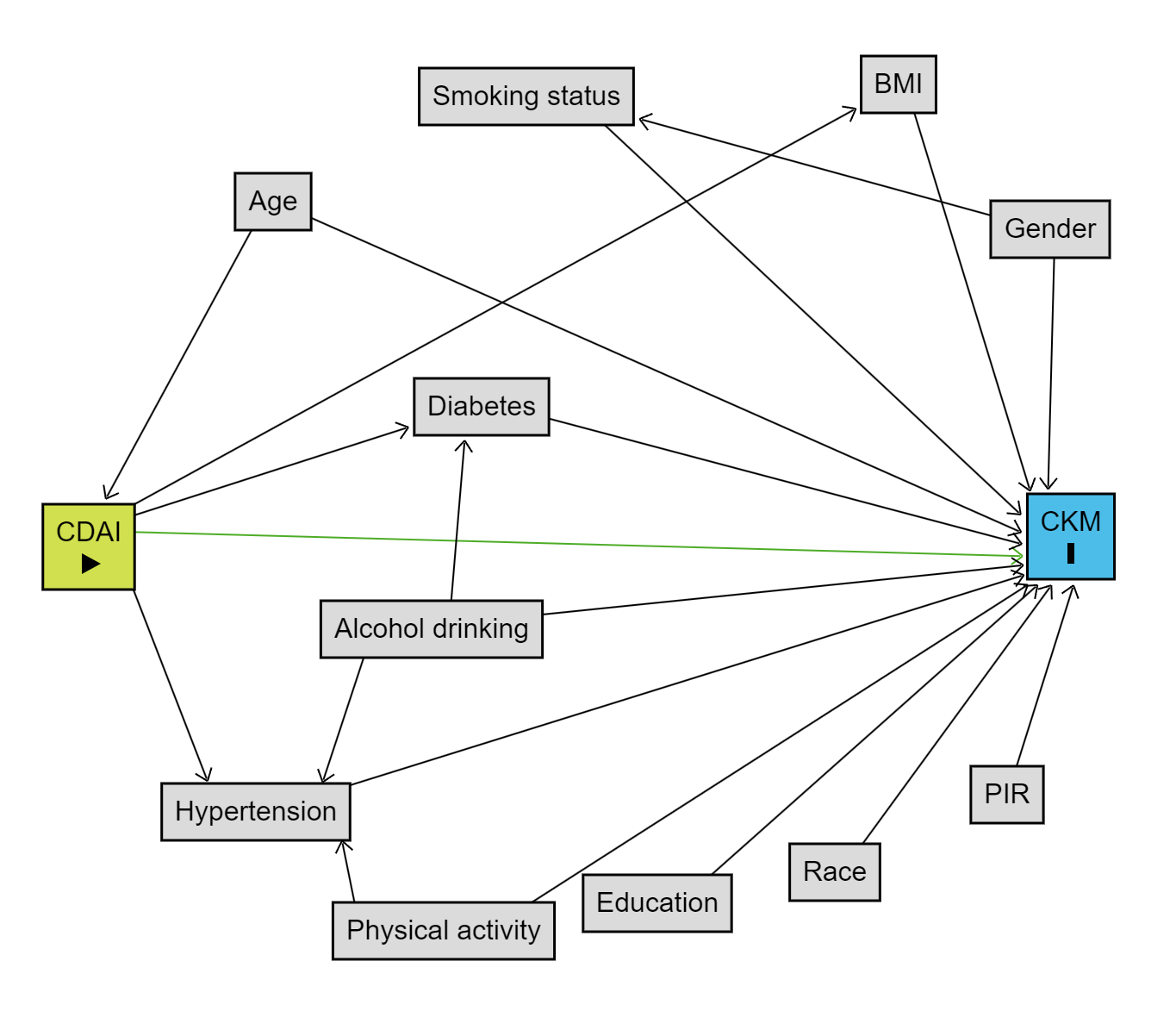


**Figure S2** Directed acyclic graph (DAG). Abbreviations: *BMI*, body mass index; *PIR*, poverty-to-income ratio.

**
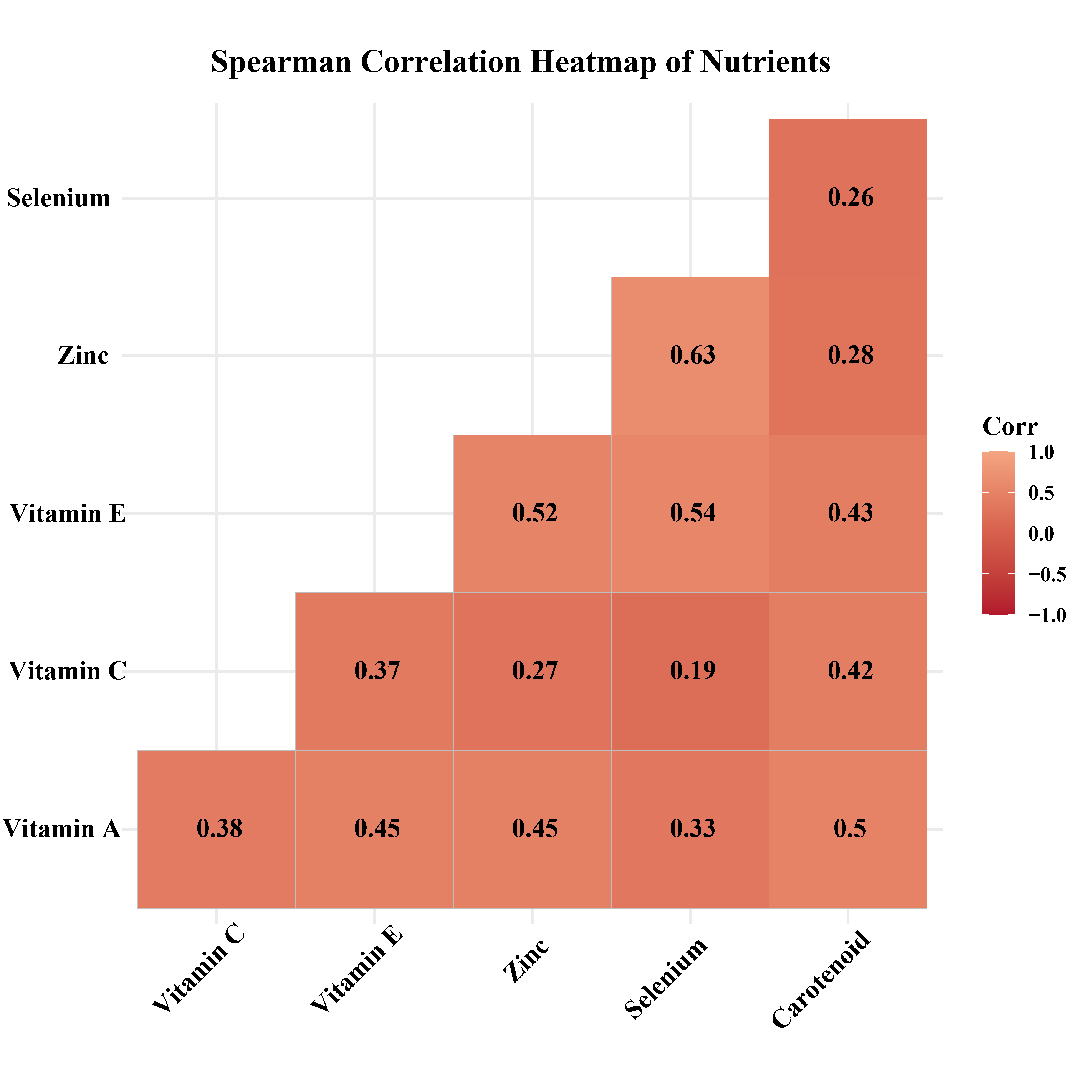
**

**Figure S3** Pairwise Spearman correlation coefficients among CDAI components among older adults in NHANES 2001 to 2020.


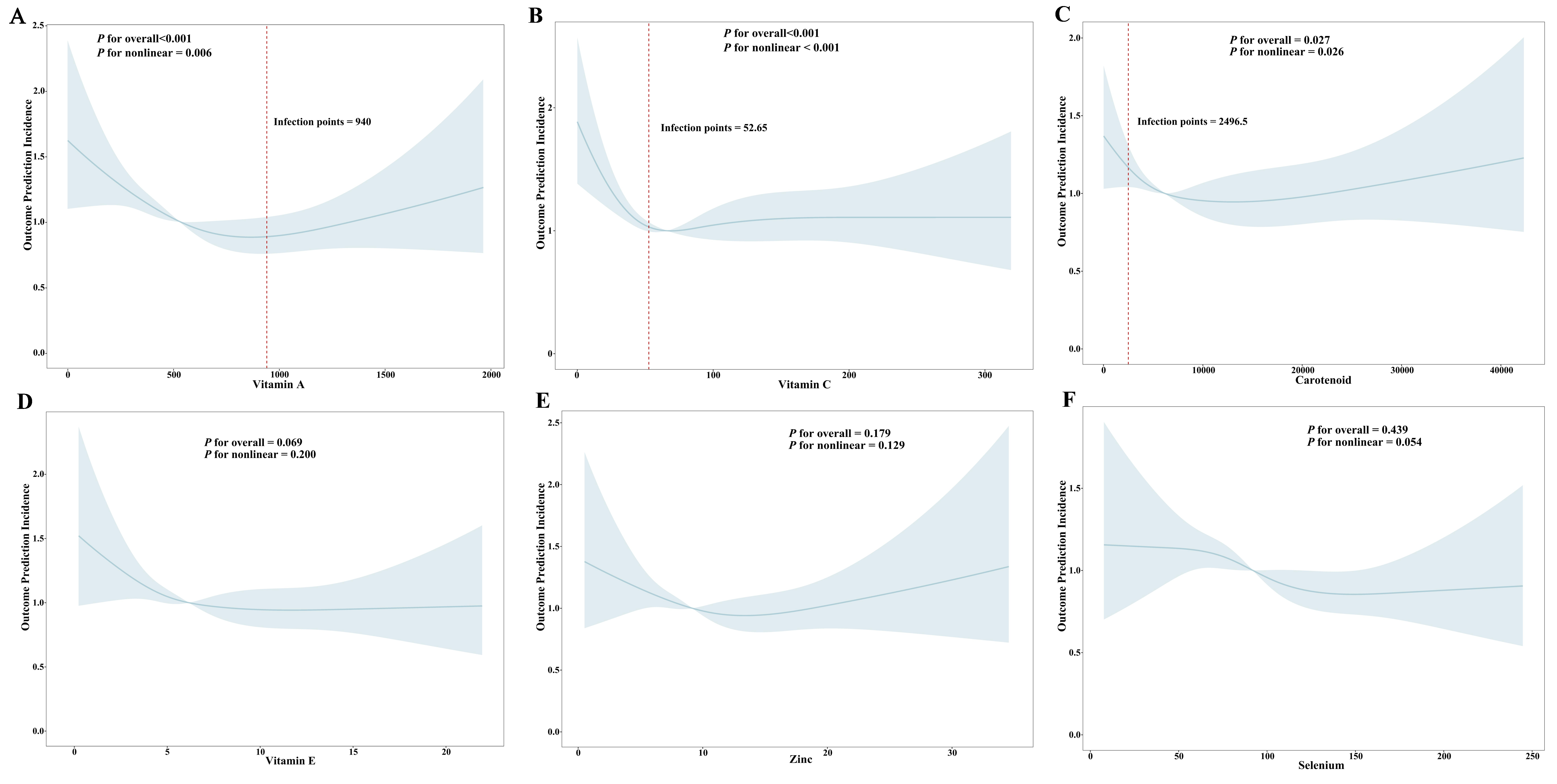


**Figure S4** Restricted cubic spline (RCS) analysis with multivariate-adjusted associations between dietary antioxidant micronutrients and odds of advanced CKM syndrome in older adults. Abbreviations: *CDAI*, composite dietary antioxidant index; *CKM*, Cardiovascular-Kidney-Metabolic

Model was adjusted for age, gender, race/ethnicity, education levels, PIR, BMI, smoking status, physical activity, alcohol intake, hypertension, and diabetes.
